# PET imaging with [^68^Ga]-labeled TGFβ-targeting peptide in a mouse PANC-1 tumor model

**DOI:** 10.3389/fonc.2023.1228281

**Published:** 2023-09-15

**Authors:** Yong Li, Hong Zhao, Shan Hu, Xichen Zhang, Haojian Chen, Qihuang Zheng

**Affiliations:** ^1^ Department of Nuclear Medicine, Shenzhen Hospital of Southern Medical University, Shenzhen, China; ^2^ Department of Nuclear Medicine, Shenzhen People’s Hospital, Shenzhen, China; ^3^ Department of Radiology and Imaging Sciences, Indiana University School of Medicine, Indianapolis, IN, United States

**Keywords:** TGFβ, targeting peptide, diagnostic tracer, PET imaging, pancreatic cancer

## Abstract

**Purpose:**

Transforming growth factor β (TGFβ) is upregulated in many types of tumors and plays important roles in tumor microenvironment construction, immune escape, invasion, and metastasis. The therapeutic effect of antibodies and nuclide-conjugated drugs targeting TGFβ has not been ideal. Targeting TGFβ with small-molecule or peptide carriers labeled with diagnostic/therapeutic nuclides is a new development direction. This study aimed to explore and confirm the imaging diagnostic efficiency of TGFβ-targeting peptide P144 coupled with [^68^Ga] in a PANC-1 tumor model.

**Procedures:**

TGFβ-targeting inhibitory peptide P144 with stable activity was prepared through peptide synthesis and screening, and P144 was coupled with biological chelator DOTA and labeled with radionuclide [^68^Ga] to achieve a stable TGFβ-targeting tracer [^68^Ga]Ga-P144. This tracer was first used for positron emission tomography (PET) molecular imaging study of pancreatic cancer in a mouse PANC-1 tumor model.

**Results:**

[^68^Ga]Ga-P144 had a high targeted uptake and relatively long uptake retention time in tumors and lower uptakes in non-target organs and backgrounds. Target pre-blocking experiment with the cold drug P144-DOTA demonstrated that the radioactive uptake with [^68^Ga]Ga-P144 PET *in vivo*, especially in tumor tissue, had a high TGFβ-targeting specificity. [^68^Ga]Ga-P144 PET had ideal imaging efficiency in PANC-1 tumor-bearing mice, with high specificity *in vivo* and good tumor-targeting effect.

**Conclusion:**

[^68^Ga]Ga-P144 has relatively high specificity and tumor-targeted uptake and may be developed as a promising diagnostic tool for TGFβ-positive malignancies.

## 1 Introduction

Transforming growth factor β (TGFβ) is a multifunctional cytokine that controls cell proliferation, differentiation, and other functions in many cell types. Dysregulation of TGFβ activation and signaling may result in cell apoptosis (1). Many cells express TGFβ, and almost all of them have specific TGFβ receptors. Members of the TGFβ family all function through the same receptor signaling systems, and by inducing the recruitment and activation of SMAD family transcription factors, they regulate cell differentiation and growth, formation of extracellular matrix (ECM), and modulation of the immune response (2–4). TGFβ also regulates the expression and activation of other important cytokines, including interferon γ and tumor necrosis factor α (5). TGFβ is commonly upregulated in tumor cells and plays an important role in tumor microenvironment (TME) construction and immune escape (6, 7). TGFβ induces M1–M2 phenotypic transformation of macrophages and N1–N2 phenotypic transformation of neutrophils in the TME and promotes matrix remodeling, angiogenesis, lymphangiogenesis, and epithelial–mesenchymal transformation (EMT) (8–10). By acting on tumor cells and immune cells in the TME, TGFβ promotes tumor immunosuppression and tumor cell invasion and metastasis. TME stromal factors including TGFβ and fibroblast activation protein are promotively linked to tumor immune escape and drug resistance (11–13). Therefore, tumor stromal factors such as TGFβ can be researched and developed as promising targets for tumor diagnosis and therapy.

At present, there are many TGFβ-based developmental strategies for targeted diagnostic and therapeutic drugs. Results of positron emission tomography (PET) molecular imaging with diagnostic nuclide-labeled tracer using TGFβ-specific monoclonal antibody GC1008 as a carrier show that [^89^Zr]-GC1008 well penetrated recurrent high-grade gliomas, but the antibody drug GC1008 itself did not achieve the expected clinical effect in terms of efficacy (14). Anti-TGFβ therapy potentiates infiltration of T cells into the tumor core. It has been well reported that TGFβ treatment contributed to T-cell exclusion and attenuated tumor response to PD-L1 inhibition, while combined therapy with TGFβ-blocking antibody and anti-PD-1 drug reduced TGFβ signaling in stromal cells, facilitated T-cell penetration into the tumor core, and provoked vigorous antitumor immunity and tumor regression (15). Small-molecule inhibitors targeting TGFβ and its receptors are also used for the therapy and diagnosis of tumors. Radionuclide-labeled small-molecule PET imaging tracers targeting the TGFβ and TGFβR1/2 signaling pathway have been investigated, such as PET tracer platforms that target TGFβR1 (ALK5) (16). Preclinical and clinical studies have shown that diagnostic and therapeutic strategies of TGFβ-targeting antibody drugs, including radionuclide-coupled drugs, are not quite satisfactory. Therefore, TGFβ-targeting small-molecule or peptide inhibitors, and more diagnostic/therapeutic nuclide-labeled drugs are further effort directions of TGFβ target development.

TGFβ is one of the main inducing factors for tumor matrix formation, playing important roles in carcinoma-associated fibroblast activation, tumor microenvironment construction, immune escape, and drug resistance (12, 17, 18). It also promotes the occurrence and development of fibrotic diseases (19, 20). Pancreatic adenocarcinoma tumors are dense solid tumors with significantly enhanced stromal connective tissue response, characterized by a high degree of fibrosis in the ECM (21–23). Previous studies have shown that as TGFβ-targeting tracers, the inhibitory small molecules or polypeptides can exert good efficiency in PET imaging for pancreatic cancer (24–26). According to the biodistribution characteristics of TGFβ *in vivo* and the solid tumor-penetrating property of TGFβ-targeting carrier drugs in existing research reports (14, 24, 25, 27), development of radionuclide-labeled targeting small-molecule or peptide inhibitors for imaging diagnostic application can be carried out, with potential indications including hematological tumors such as lymphoma, glioblastoma, sarcoma, pancreatic adenocarcinoma, lung squamous cell carcinoma, hepatocellular carcinoma, renal cell carcinoma, breast cancer, colorectal cancer, and urinary system cancers.

In this study, we planned to investigate and examine the tumor diagnostic efficiency of TGFβ-targeting inhibitory peptide P144 coupled with [^68^Ga]. The peptide P144 was radiolabeled with [^68^Ga] to form a stable TGFβ-targeting tracer [^68^Ga]Ga-P144, which was used for diagnostic PET imaging verification in the pancreatic cancer PANC-1 tumor model. The significance of this study is to develop a high-specific TGFβ-targeting small-molecule peptide inhibitor and radionuclide-labeled diagnostic drug. Developmental strategies of tumor diagnosis and treatment with antagonistic peptides such as P144 as targeted small-molecule carriers may become a promising direction in TGFβ target-related cancer research.

## 2 Materials and methods

### 2.1 Animals and agents

Animal experimental procedures were performed in accordance with the National Research Council’s Guide for the Care and Use of Medical Laboratory Animals (Ministry of Health, China). All the animal experimental protocols were approved by the Institutional Animal Care and Research Ethics Committee of Shenzhen Hospital of Southern Medical University. Male NOD-SCID mice aged 6–8 weeks and healthy male SD rats aged 8–10 weeks (200 ± 10 g) were obtained from Beijing Vital River Laboratory Animal Technology Co., Ltd. (Beijing, China). All mice were kept in a specific pathogen-free (SPF)-grade animal house under 12-h light/dark cycles with controlled temperature (24°C ± 2°C) and relative humidity (50%–60%) and were provided with a standard rodent chow diet and water *ad libitum*. When used for experiments, the average weight of the mice was 22 ± 2 g, and the age was approximately 12 weeks. Pancreatic cancer cell line PANC-1 was obtained from the American Type Culture Collection (ATCC), pentobarbital sodium was purchased from Sigma-Aldrich (Darmstadt, Germany), rabbit anti-TGFβ monoclonal antibody (#3711S) was purchased from Cell Signaling Technology (Danvers, MA, USA), and secondary antibodies and Hoechst 33258 staining kit were obtained from Beyotime Biotechnology (Shanghai, China).

### 2.2 Probe synthesis and quality control

TGFβ-specific blocking tetradecapeptide P144 is a fragmental analog of the extracellular domain of TGFβR3 (28), and its synthesis was entrusted to a third-party organization company (Hangzhou Chinese Peptide Company, Hangzhou, China). P144-DOTA (molecular weight, 2,114.4 Da) was obtained by coupling P144 with the biological chelator DOTA through a polyethylene glycol short chain (n = 3), and the high-performance liquid chromatography (HPLC) method was used for quality control. Then, P144-DOTA was labeled with radionuclide [^68^Ga]. A radiolabeling module (Smart Module-X) was designed and developed for the synthesis of radionuclide-labeled tracers. The radiolabeling synthesis method for [^68^Ga]Ga-DOTA-P144 was conducted based on a similar methodology as previous reports with modifications (29). The ^68^Ge/^68^Ga generator was rinsed with 0.1 M of HCl, and the pH value of the rinsing solution was adjusted to between 3.5 and 4.5 with 1.25 M of acetic acid sodium buffer. A volume of 200 μL (200 μg) of the precursor was taken after it was mixed evenly, and then it was added into a volume of 3 mL of rinsing solution (radioactivity of ^68^Ga was approximately 370 MBq, that is, 10 mCi), and it was heated in a constant-temperature metal bath at 100°C for 10 min. After cooling, 20 mL of sterile water was added for dilution, and the liquid was passed through the C18 Sep-Pak light solid-phase extraction column and rinsed with 5 mL of sterile water for injection to remove free ^68^Ga ions, ^68^Ge ions, and water-soluble impurities. Then, it was eluted with 10 mL of 75% ethanol, and the ethanol solvents were removed from the radiolabeled compound solution using a nitrogen stream. Finally, the sterile filter membrane was placed into the sterile sealed bottle, and sterilizing filtration of [^68^Ga]Ga-DOTA-P144 product solution was performed before injection.

Radio-HPLC and radio-iTLC assays were conducted for the quality control of product [^68^Ga]Ga-DOTA-P144 and free [^68^Ga] ions. American alltech1500 high-performance liquid chromatography and online radioactive detector were used. Chromatographic column: YMC-Pack Pro C18 RS (5 μm, 250 mm × 4.6 mm). Mobile phase: A) water + 0.1% formic acid; B) acetonitrile. Method: 0 min, 95% A and 5% B; 1 min, 95% A and 5% B; 10 min, 70% A and 30% B; 18 min, 70% A and 30% B; 25 min, 95% A and 5% B; flow speed, 1 mL/min. The quality control standard was that the chemical and radiochemical purities of the products were greater than 95%. Radio-iTLC analytical quantification methods/conditions were as follows. The stationary phase was iTLC glass fiber (1 cm × 8 cm instant silica gel strip), and the developing solvent was 2 mM of sodium citrate/citric acid buffer solution (pH 6.5). performed according to General Rule 1401 in Chinese Pharmacopoeia (2020 Edition), the method for the determination of radioactive drugs, the Rf value of the radioactive peak of the product to be tested was approximately 0.1–0.4, and there was a single main peak under normal conditions. If there were impurities, especially free [^68^Ga] ions, the corresponding peaks should appear at the Rf value of approximately 0.6–0.8.

### 2.3 Cell line and tumor model

Pancreatic cancer cell line PANC-1 was obtained from the ATCC, and cells were cultured in complete RPMI1640 medium supplemented with 10% fetal calf serum (Life Technologies, Carlsbad, CA, USA). One portion of the PANC-1 cells (~1 × 10^6^) was injected subcutaneously below the anterior axillary of the nude mice to induce the mouse heterotopic tumor model. The tumor cells were suspended in a mixture (v/v, 1:1) of medium and Matrigel (Corning Life Sciences, Corning, NY, USA). The tumor xenografts were generally palpable within 10 days after injection. Xenograft tumor sizes of the model mice were measured every 3 days. Short and long tumor diameters, tumor volume, and body weight were measured. When the xenograft tumor grew to a volume of 150–200 mm^3^, the mice were used for PET imaging study. A diagnostic tracer and drug were injected approximately 2 weeks after the establishment of the tumor model.

### 2.4 Cell uptake assay

The method process for cell uptake assay of radiolabeled drug was as follows. First, the selected cell lines were inoculated into a 6-well plate, incubated for 48 h to achieve a cell coverage rate of approximately 80%–90% (1.2–2 × 10^6^ cells/well), and replaced with 1 mL of fresh culture medium without fetal calf serum (FCS). Then, a certain amount of radionuclide-labeled drug (200 MBq/L) was added to the culture well and incubated for different times (0.5–5 h). The culture medium containing the drug was removed, and the cells were washed twice with 1 mL of phosphate-buffered saline (PBS) (pH 7.4). Cells were incubated with 1 mL of glycine HCl solution (1 M, pH 2.2) at room temperature for 10 min to remove surface binding, and the cells were washed with 2 mL of ice-cold PBS (collect washing solution for detection of binding amount). After that, 1.4 mL of lysis buffer (0.3 M of NaOH, 0.2% sodium dodecyl sulfate (SDS)) was used to lyse the cells and collect this lysate. With a γ-counter, the radioactive activity in the collected lysate was measured, and the %AD value was calculated. One mio (1 × 10^6^) of cells was adopted for normalization, with the result representing the internalization uptake level of the tested radiopharmaceutical by this cell line. Each experiment was repeated three times independently, with three multiple wells set at each processing time point.

### 2.5 Immunohistochemistry

Tumor tissues and normal tissues were isolated and immersed in 10% neutral formalin. After dehydration in 30% sucrose, the tissue block was embedded in paraffin and cut into slices with a thickness of 5 μm. The tissue slices were blocked with 3% bovine serum albumin (BSA) in PBS and incubated with a concentration of 2 μg/mL of primary antibody (anti-TGFβ-1 mouse monoclonal antibody, TB21, Thermo Fisher Scientific, Waltham, MA, USA) at 4°C overnight. After washing, the horseradish peroxidase (HRP)-conjugated goat anti-mouse IgG secondary antibody (Beyotime Biotechnology, Shanghai, China) (1:400 dilution in volume) was incubated for 1 h at room temperature and washed with PBS three times. After coloration and washing, slices were mounted on glass slides, sealed with 30% glycerin, and visualized under inverted microscopy (Olympus IX71, Olympus Co., Tokyo, Japan).

### 2.6 Drug administration

Peptide conjugate P144-DOTA and the radiolabeled tracer [^68^Ga]Ga-P144 were both administrated through tail vein injection. The chemical dose of [^68^Ga]Ga-P144 was 1 mg/kg, and the radioactive dose was approximately 3.7 MBq in 200 μL per mouse. As a competitive blocker with the same target TGFβ, the dose of DOTA-P144 was 100 mg/kg (47.3 μmol/kg, 100 times the mass dose of the radiolabeled peptide conjugate), and the administration of this unlabeled peptide conjugate was followed after 10 min by 1 mg/kg of the radiolabeled tracer [^68^Ga]Ga-P144. All the mice were deprived of food and water for 1 h before drug administration. Then, the mice were anesthetized with pentobarbital sodium (45 mg/kg) and scanned with microPET. After the last scanning, the mice were anesthetized with 2% pentobarbital sodium at 65 mg/kg intraperitoneally and then executed by dislocation of their cervical vertebra, and the tissues were harvested for analysis.

### 2.7 MicroPET imaging

The tumor-bearing mice were anesthetized with pentobarbital sodium before microPET scanning. Each mouse was intravenously injected with approximately 3.7 MBq (90–120 μCi) of [^68^Ga]Ga-P144. After administration, the mice were scanned by a small animal PET system (Inveon, Siemens, Munich, Germany). The body temperature of the mice was monitored by a rectal probe and kept at 37°C by a heated air stream. The scanning time was set as follows: static PET scanning for 10 min and scanning energy window 350–650 keV at four time points (1 h, 2 h, 3 h, and 4 h) after the injection. MicroPET image reconstruction was performed using a 3D OSEM PSF algorithm with five iterations. Images were processed and analyzed using PMOD4.2 software (PMOD Technologies Ltd., Zurich, Switzerland). Regions of interest (ROIs) of the brain, heart, liver, lungs, kidney, muscle, and tumor were delineated. Radioactivity values of the ROIs per unit volume were obtained, and the percentage of injected dose per tissue weight in gram (%ID/g) values of the different organs and tissues were calculated as follows:


$$\%\text{ID}/\text{g}=\frac{\text{radiouptake in ROI}(\frac{\text{kBq}}{\text{g}})}{\text{injection dose}(\text{kBq})}\times 100.$$

### 2.8 Biodistribution assay

For biodistribution assay *ex vivo*, the PANC-1 tumor model mice were euthanized by pentobarbital sodium at 1 h after tracer administration. The blood was harvested via cardiac puncture, and the different organs/tissues were isolated, weighed, and counted on a γ-counter for radioactivity. The amount of the injected radiotracer was measured and used to determine the total number of counts of nuclei decay per minute (CPM) by comparison with a standard of known activity. The data were background- and decay-corrected and expressed as percentages of the injection dose per tissue weight in grams (%ID/g).

### 2.9 Statistical analysis

Data analysis was performed using SPSS19.0 software (SPSS Inc., USA), and the quantitative data were presented as “mean ± SD”. Differences between groups were compared using a two-tailed independent samples t-test. A difference at a p-value below 0.05 was considered statistically significant.

## 3 Results

### 3.1 Synthesis and radiolabeling of DOTA-P144 and quality control

**Figure 1:**
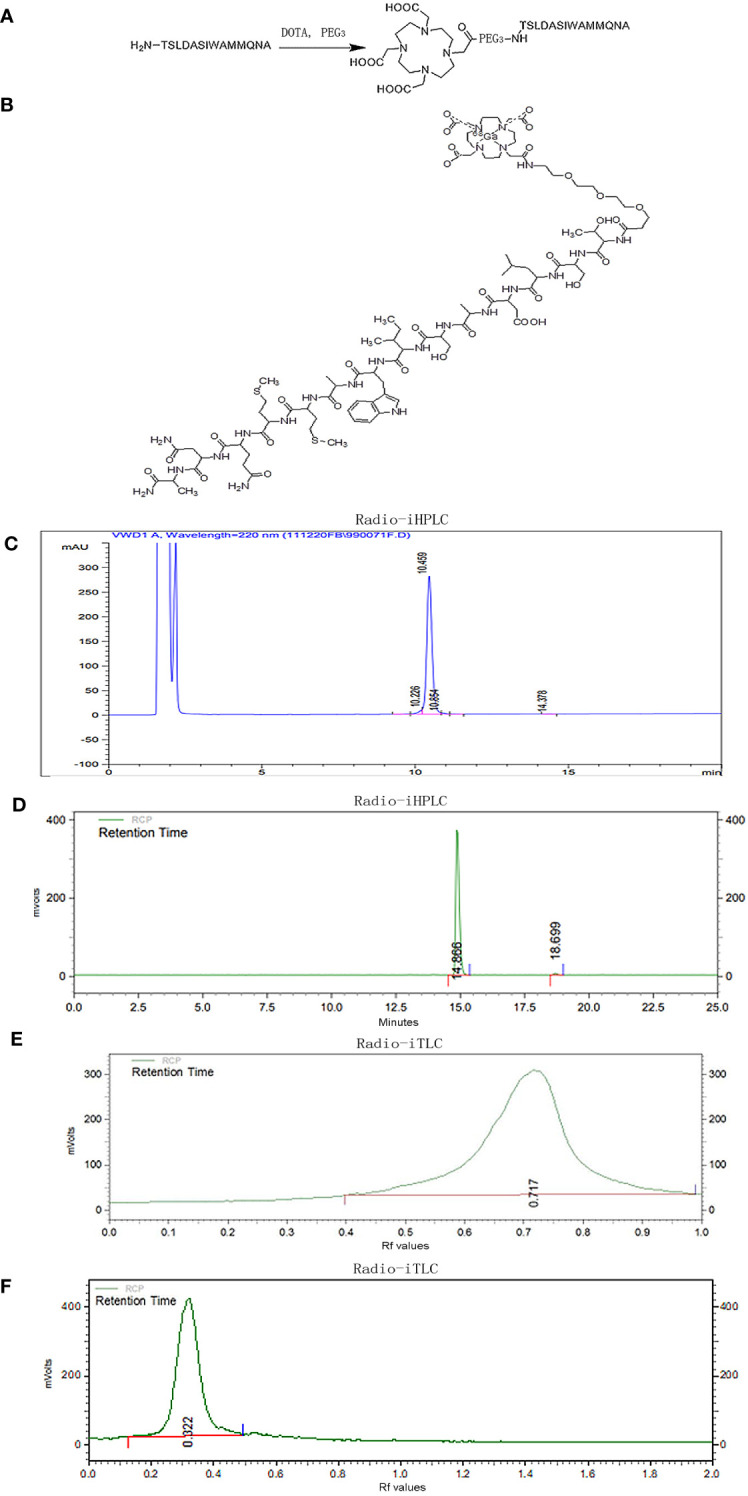
Preparation and quality control of radiotracer [^68^Ga]Ga-P144. **(A)** Schematic chart of connection of chelator DOTA to TGFβ-specific tetradecapeptide P144. **(B)** Structure of radiolabeled DOTA-P144 with diagnostic nuclide [^68^Ga]. **(C)** Representative HPLC chromatogram of unlabeled DOTA-P144. **(D)** Radio-HPLC chromatogram of the radiolabeled tracer [^68^Ga]Ga-P144 at 4 h after synthesis. **(E)** Radio-iTLC chromatogram of the free [^68^Ga^3+^] ions. **(F)** Radio-iTLC chromatogram of [^68^Ga]Ga-P144 showing the Rf value and high radiochemical purity of the radioactive nuclide-labeled product [^68^Ga]Ga-P144. HPLC, high-performance liquid chromatography.

Based on the extracellular domain fragment of TGFBR3, we focused on a series of candidate antagonistic peptides targeting TGFβ, including pentadecapeptide P17 (KRIWFIPRSSWYERA) and tetradecapeptide P144 (TSLDASIWAMMQNA). We finally chose P144 for specific targeting and binding to TGFβ considering its good performance in a study with human glioblastoma cell lines (28). As shown in **Figure 1A**, the connection of P144 to chelator DOTA was as follows: the free amino group on the threonine residue at one end of P144 and a carboxyl group of DOTA was connected by a covalent bond through a flexible short chain of ethylene glycol. Radioactive nuclide [^68^Ga] was labeled to P144 peptide by the connected chelating agent DOTA (**Figure 1B**). After separation and detection by the radio-HPLC system, the retention time of the conjugated peptide DOTA-P144 (molecular weight = 2,114.4 Da) was 10.459 min, and the chemical purity represented by peak area percentage was 96.401% (>95%; **Figure 1C**). As shown in **Figure 1D**, the radiochemical purity of the nuclide-labeled product [^68^Ga]Ga-P144 compound was 98.87% after purification and placement at room temperature for 4 h, and the retention time was 14.866 min. By radio-iTLC analysis, we found that the Rf value of free ^68^Ga^3+^ ions was 0.717, and the purity was 100% (**Figure 1E**). The Rf value of [^68^Ga]Ga-P144 in the radio-iTLC chromatogram was 0.322, and the radiochemical purity was 100% (**Figure 1F**).

### 3.2 Distribution of TGFβ in pancreatic cancer PANC-1 mouse model

**Figure 2:**
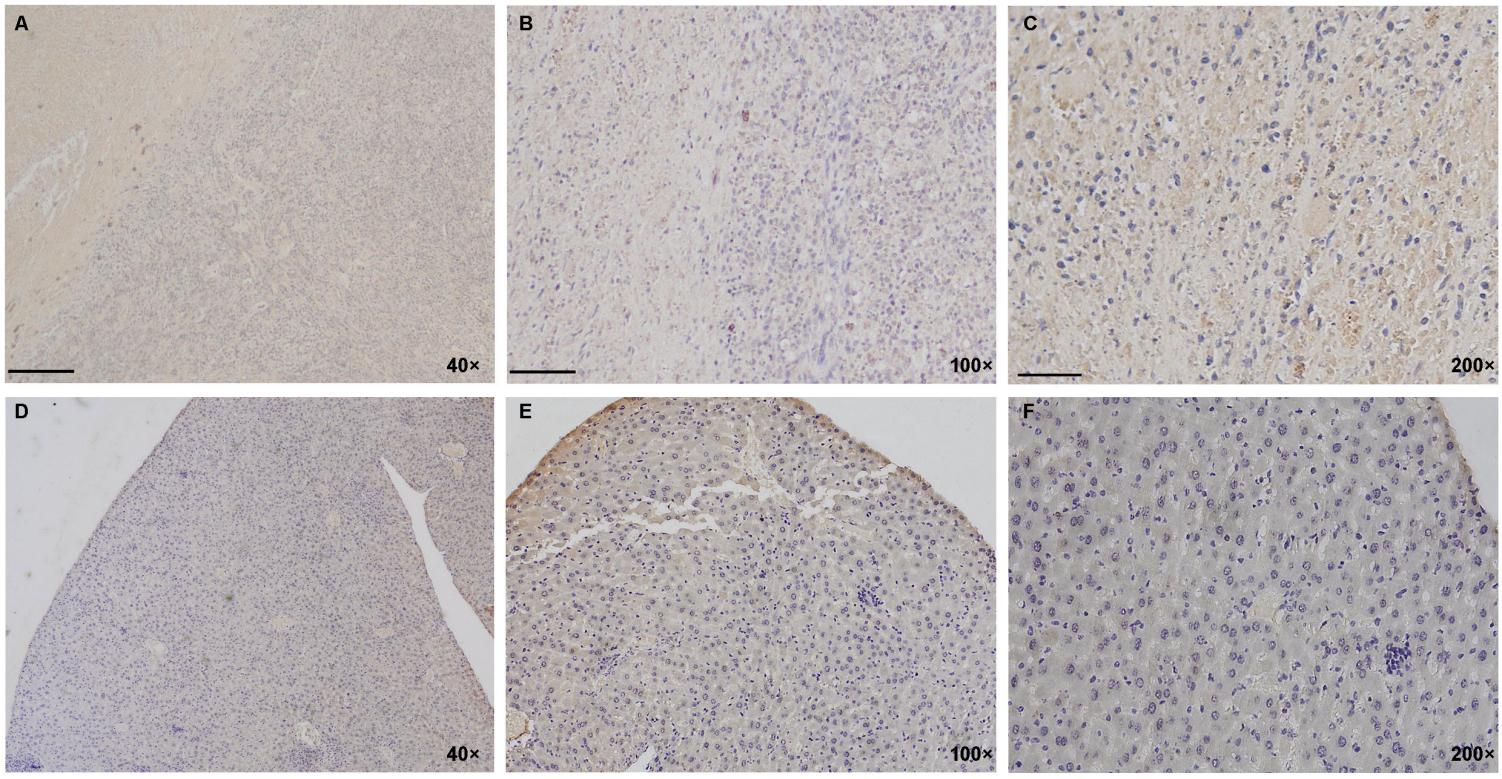
Distribution of TGFβ in PANC-1 tumor model of mice. **(A–F)** IHC staining results show a high positive distribution of TGFβ in the PANC-1 tumor tissue (above lane, **A–C**, yellow and brown) while negative or very low basal expression of TGFβ in non-tumor tissue (normal liver tissue; below lane, **D–F**). Dark blue, cellular nuclei stained with Hoechst 33258. Images in the left lane were magnified 40 times, in the middle lane 100 times, and in the right lane 200 times. Scale bars, left, 100 μm; middle, 40 μm; right, 20 μm. IHC, immunohistochemistry.

In the human PANC-1 cell line-established mouse pancreatic cancer animal model, immunohistochemical assay was performed to evaluate and confirm the expression and distribution of TGFβ in tumor model tissues. The PANC-1 tumor-bearing nude mice with a xenograft tumor volume over 1,000 mm^3^ were sacrificed, and the tumor tissue and normal liver tissue were isolated for immunohistochemistry (IHC) assay with an antibody against TGFβ. Hoechst 33258 stain solution was used as a staining indicator for cellular nuclei in the IHC assay. As shown in **Figures 2A–F**, the IHC result demonstrated the high-intensity distribution of TGFβ in tumor tissue of the mouse model of PANC-1 pancreatic cancer, while the expression of TGFβ in non-tumor tissue (normal liver tissue) was at a very low level.

### 3.3 [^68^Ga]Ga-P144 used for microPET imaging in mouse PANC-1 tumor model

**Figure 3:**
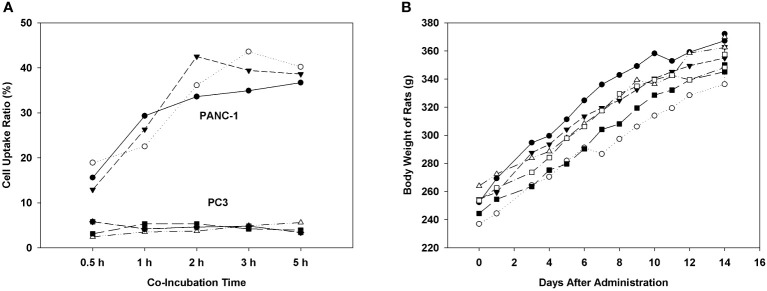
Cellular uptake and biosafety of [^68^Ga]Ga-P144. **(A)** Results of cellular uptake assay of [^68^Ga]Ga-P144 in PANC-1 and PC3 cell lines show a high and stable internalization rate (cell uptake ratio) of [^68^Ga]Ga-P144 in PANC-1 cells and a very low level of non-specific uptake in PC3 cells (three series of cell assays for each cell line). **(B)** Body weights of rats on different days after the administration of [^68^Ga]Ga-P144 (500 μCi per rat, six rats) displaying normal rapid weight growth during the exposure period to a relatively high level of [^68^Ga]Ga-P144 for at least 14 days.

We examined the binding and internalization capability of [^68^Ga]Ga-P144 in PANC-1 cells and tested the *in vivo* safety of [^68^Ga]Ga-P144 in normal male SD rats. As shown in **Figure 3A**, cellular uptake assay results (six series of cell assays) of [^68^Ga]Ga-P144 with PANC-1 and PC3 cell lines showed high and persistent cell uptake ratio of [^68^Ga]Ga-P144 in PANC-1 cells and very low level of non-specific cellular uptake in PC3 cells. **Figure 3B** shows the body weight growth changes of six healthy rats on different days after the administration of the radiopharmaceutical [^68^Ga]Ga-P144 (500 μCi per rat), and the rapid body weight growth of all tested rats that were exposed to a relatively high dose of [^68^Ga]Ga-P144 for at least 14 days after administration indicated good biosafety *in vivo* for the development of this radiotracer and its further clinical study.

**Figure 4:**
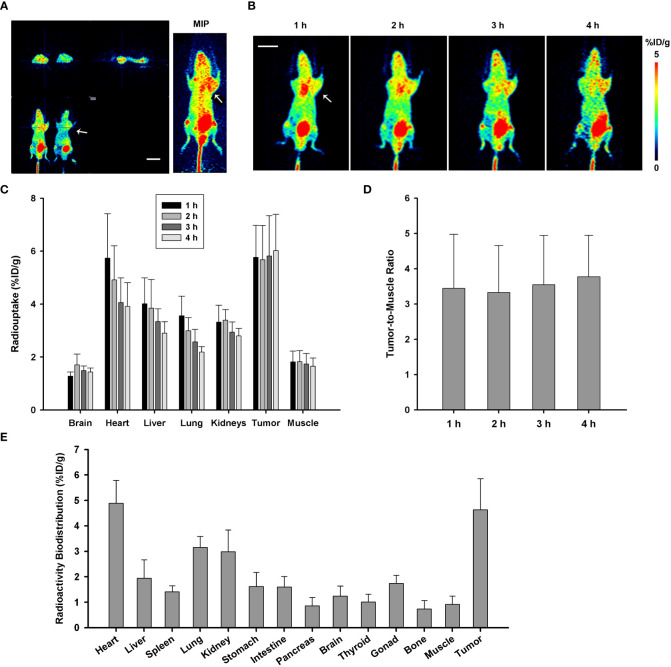
MicroPET imaging and analysis with [^68^Ga]Ga-P144 in PANC-1 model of mice. **(A)** Representative microPET images with radiotracer [^68^Ga]Ga-P144 in PANC-1 tumor model mice; three different sections with tumor location and MIP image at 3 h post-injection. The small arrow indicates the location of the xenograft tumor. Scale bar, 1 cm. **(B)** Representative coronal section images at four time points post-injection showing relatively high radioactive uptake in tumor areas compared with normal tissues. The small arrow indicates the location of the xenograft tumor. Scale bar, 1 cm. **(C)** Statistical histogram showing the radioactive uptake values in main normal organs and tumor quantified at different time points (n = 5). Data were expressed as “mean ± SD”. **(D)** Statistical histogram showing the average tumor-to-muscle ratio (TBR) of radioactive uptake at the four time points after tracer injection (n = 5). Data were expressed as “mean ± SD”. **(E)** *Ex vivo* radioactive biodistribution of [^68^Ga]Ga-P144 in PANC-1 tumor model mice at 1 h after tracer injection (n = 5). Data were expressed as “mean ± SD”.

PANC-1 xenograft tumor model of pancreatic cancer in mice was built to investigate the imaging efficiency of P144-based conjugate [^68^Ga]Ga-P144. The mice were injected with approximately 3.7 MBq of [^68^Ga]Ga-P144 (1 mg/kg) via a caudal vein, and microPET scans were performed at 1 h, 2 h, 3 h, and 4 h after tracer injection. Representative [^68^Ga]Ga-P144 microPET images (**Figure 4A**, different section images with tumor location and MIP image at 3 h after injection; **Figure 4B**, coronal section images at four time points) indicated higher radioactive uptake in tumor areas compared with normal tissue. Radioactive uptake in main organs and tissues was quantified at different time points (**Figure 4C**). Uptakes in the heart (%ID/g-mean, 5.738 ± 1.681), liver (4.014 ± 0.975), lung (3.560 ± 0.739), kidneys (3.322 ± 0.636), and tumors (5.764 ± 1.212) were all relatively high at 1 h after injection. At 4 h post-injection, radioactive distribution in the heart (%ID/g-mean, 3.919 ± 0.891), liver (2.906 ± 0.431), lung (2.190 ± 0.206), and kidneys (2.804 ± 0.281) slightly but continuously decreased. However, uptake in tumors slightly increased (%ID/g-mean, 6.023 ± 1.370) (n = 5 mice per time point). As shown in **Figure 4D**, the tumor-to-muscle ratio also slightly increased from 3.442 ± 1.531 at 1 h to 3.770 ± 1.178 at 4 h. These results indicated that [^68^Ga]Ga-P144 has good targeting to TGFβ-positive tumors, and it may transfer and redistribute from the heart, liver, and lung to tumor tissues *in vivo* and has an adequate long retention time and good targeting efficiency for PANC-1 tumor. In addition, as shown in **Figure 4E**, [^68^Ga]Ga-P144 radioactive distribution *ex vivo* in different isolated organs/tissues of PANC-1 tumor model mice (n = 5) at 1 h after tracer injection displayed considerable consistence with the result of *in vivo* uptakes, with relatively high radioactive biodistributions in the heart (4.886 ± 0.899), liver (1.948 ± 0.717), lung (3.156 ± 0.429), kidney (2.985 ± 0.854), and tumors (4.635 ± 1.217).

### 3.4 TGFβ-specific targeting confirmation by microPET with unlabeled P144

**Figure 5:**
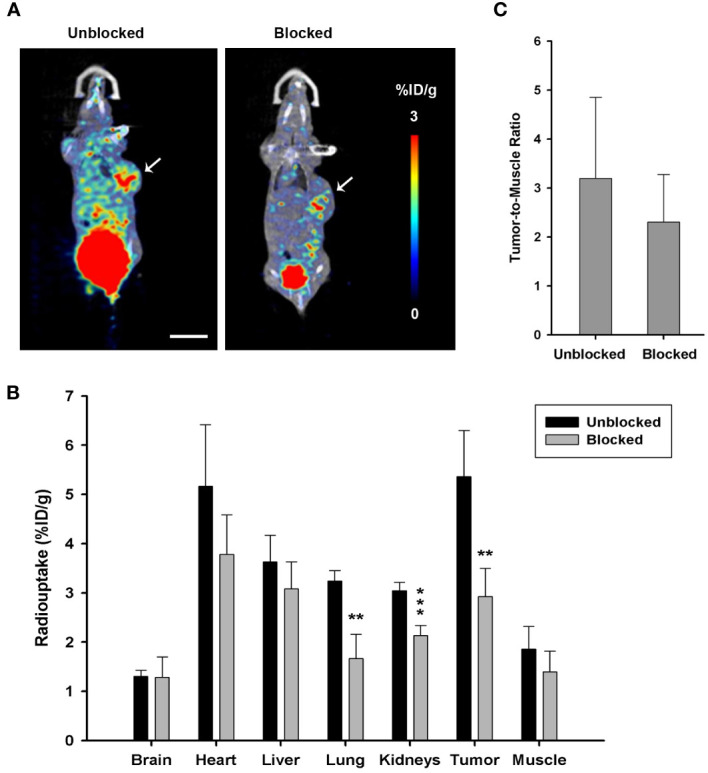
Target blocking study via microPET imaging with unlabeled DOTA-P144. **(A)** Representative microPET/CT coronal section images with radiotracer [^68^Ga]Ga-P144 alone (unblocked) or [^68^Ga]Ga-P144 following a 100-fold mass dose of unlabeled DOTA-P144 for blockade (blocked) in mouse PANC-1 tumor model at 1-h time point after radiotracer injection. Small arrows indicate the location of the xenograft tumor. Scale bar, 1 cm. **(B)** Statistical histogram showing the radioactive uptake values in the main organs and tumor of the unblocked and blocked groups (n = 4). Data were expressed as “mean ± SD”. Two-tailed independent samples t-test was used. **p< 0.01, ***p< 0.001 *vs.* unblocked group. **(C)** Statistical histogram showing the average tumor-to-muscle ratio (TBR) of radioactive uptake at 1-h time point after tracer injection (n = 4). Data were expressed as “mean ± SD”.

MicroPET imaging in the PANC-1 tumor model for competitive TGFβ blockade study was performed with unlabeled DOTA-P144 pretreatment (100 times the mass dose of [^68^Ga]Ga-P144) for 10 min before the injection of PET tracer [^68^Ga]Ga-P144 to examine and verify the targeting specificity of P144 carrier against TGFβ. As shown in **Figures 5A, B**, tumor uptake (%ID/g-mean) of [^68^Ga]-P144 in the blocked group (1 h, 2.927 ± 0.572, p = 0.004398 *vs.* unblocked) was significantly lower than that of the unblocked group (5.362 ± 0.937). When compared with those in the unblocked group, radioactive uptake values in the kidneys (unblocked, 3.045 ± 0.169; blocked, 2.133 ± 0.206) and lung (3.240 ± 0.214; 1.666 ± 0.496) of the blocked group were significantly decreased (p = 0.000475 and 0.001123, respectively). The radioactive uptakes in other organs were all slightly decreased but without significant differences (p > 0.05, n = 4 per group) between the two groups at the 1-h time point. As shown in **Figure 5C**, the tumor-to-muscle ratios of radiouptake between the unblocked and blocked groups showed no significant difference (p = 0.3867). These results confirmed the specific targeting of P144-based conjugates to TGFβ, and molecular imaging tracer [^68^Ga]Ga-P144 targeting TGFβ has potential diagnostic value for pancreatic ductal adenocarcinoma.

## 4 Discussion

In this study, we mainly used the TGFβ-specific inhibiting peptide P144 for the coupling with chelator DOTA and radiolabeling with diagnostic nuclide [^68^Ga], and the synthesis product was determined for quality control to confirm the acquisition of a radiolabeled peptide drug with high purity and stability. TGFβ-targeting radiotracer [^68^Ga]Ga-P144 was used for microPET imaging study in TGFβ-positive PANC-1 tumor-bearing models. [^68^Ga]Ga-P144 PET showed relatively high radioactive uptake and long retention in tumors and low uptake in non-target organs and backgrounds. Through target-blocking experiment with unlabeled precursor P144-DOTA, it was found that tissue radioactive uptake, especially in the tumors, as shown in [^68^Ga]Ga-P144 PET imaging results, was highly specific for the TGFβ target. This study demonstrates that [^68^Ga]Ga-P144 PET has good target specificity and targeted uptake in PANC-1 tumors, and it has potential value for development as a diagnostic tool. Research of theranostic radiopharmaceuticals with peptide antagonists such as P144, as targeted small-molecule carriers, may become an advantageous strategy for clinical development and application of TGFβ target signaling.

Through acting on tumor cells and immune cells and regulating the synthesis and release of other cytokines, TGFβ induces T-cell immunosuppression, promotes tumor immune escape, activates fibroblasts, and causes ECM remodeling and other pathological changes. TGFβ family of cytokines achieves functional homeostasis via delicate balance and crosstalk with complex signaling pathways. Inappropriate activation or inhibition of TGFβ signaling, and pathway component mutations are related to diseases such as cancers and vascular and developmental disorders (30, 31). Radiolabeled small-molecule tracers targeting TGFβ signaling components including the targets of TGFβ, TGFβ receptors, and downstream transcriptional factors SMADs can provide quantitative PET imaging for multiple useful regulators and give insights into the pathophysiological role of this pathway *in vivo*. PET imaging can also be used as a valuable method to study the drug targeting of this pathway and to detect and diagnose diseases in which this pathway is disturbed (16).

The biological functions of TGFβ may be different *in vitro* and *in vivo* and affected by the target cell states, the interaction of the cells with ECM components, and the presence or absence of other cells in the ECM. TGFβ regulates the balance between epithelial tissue and the ECM and increases the deposition of collagen and other ECM proteins by directly stimulating the expression of these genes and inhibiting the synthesis of collagenases (32). TGFβ can be expressed and released by cancer cells, stromal fibroblasts, and other cell types in the TME, further promoting cancer development, forming the system structure of the tumor, inhibiting the activities of antitumor immune cells, and consequently resulting in an immunosuppressive environment, which prevents or weakens immunotherapy efficacy (6, 7, 11, 13, 17, 33, 34). Nowadays, there are increasing preclinical and clinical studies about the diagnostic and treatment strategical development of targeted inhibitory antibody drugs and nuclide-coupled drugs (antibody-drug conjugates (ADCs)). For example, [^89^Zr]-labeled TGFβ antibody was used for immunoPET imaging of glioma (14), and a PET imaging study with [^89^Zr]-labeled TGFβ antibody fresolimumab was conducted for breast cancer (35). Results of a PET imaging study with TGFβ-specific monoclonal GC1008, as a carrier of radionuclide-labeled tracer, showed that [^89^Zr]-GC1008 could well penetrate recurrent high-grade gliomas (14). However, the clinical efficacy of simple antibody drugs targeting TGFβ is not ideal. Therefore, new TGFβ-targeting small-molecule or peptide inhibitors, as well as diagnostic and therapeutic nuclide-labeled drug candidates, may have high druggability. It has been shown that CD4-targeted antibody coupled with TGFβ-neutralizing polypeptide (similar to TGFβR2 extracellular domain) can restore Th cell antitumor immunity (33). TGFβ inhibitory peptide P144 has been designed to directly bind active TGFβ1 and block the biological effects. P144 is a hydrophobic peptide based on the extracellular sequence of human TGFβR3 and was initially researched for therapy of liver fibrosis. Previously, evidence has shown that P144 can enhance the efficacy of antitumor immunotherapy in thymoma and melanoma cell lines, and it has been proposed that P144 can act as an immunomodulator in cellular responses to tumors (28, 36).

TGFβ-specific blocking peptide P144 selected in this study was an analog of the essential structure for ligand recognition and binding in the extracellular domain of TGFβR3, which has high specificity and affinity to TGFβ (36). Studies have shown that P144 specifically blocks TGFβ signaling and could significantly inhibit the growth and proliferation of human glioblastoma cells (28). Our quality control detections showed that both P144-DOTA and its radionuclide-labeled product [^68^Ga]Ga-P144 had high purity and stability to meet the requirements of *in vivo* microPET imaging. The doses of the cold drug and radioactive drug were determined by referring to previous reports and our research experience, and the used radioactive dose was relatively low, ensuring the biosafety of this radiopharmaceutical. The findings of this study showed that, in [^68^Ga]Ga-P144 PET imaging, the background uptake of the tracer was low, the targeted uptake in the tumor was high (higher than that of main organs, see **Figure 4**), and the tumor retention time was long (>4 h), showing that [^68^Ga]Ga-P144 PET has potential diagnostic efficiency for tumor imaging.

There are still some weaknesses of this radiotracer in the present study. For example, the excretion rate of the tracer [^68^Ga]Ga-P144 from major organs including the heart, liver, lung, kidneys, muscle, and brain was relatively low. This tracer was possibly retained in the blood circulation in the early stages. Radioactive uptake in the brain was even higher than uptakes in the normal pancreas, thyroid, and bone. This finding may be unusual for a peptide-based radiotracer with a molecular size of over 2,000 Da. Therefore, further research is needed and has been planned to determine whether the ^68^Ga-labeled peptide radiotracers could cross the blood–brain barrier or just remain in blood circulation and persist with slow excretion for a long time.

We have chosen the known TGFβ-targeting peptide inhibitor P144 as a drug carrier, which has high specificity and affinity, high TGFβ signal blocking, and *in vitro* antitumor effects (28). Thus, in this study, only the *in vivo* imaging verification in tumor models was conducted, and the affinity detection of radionuclide-labeled tracer *in vitro* had not been carried out. Currently, many preclinical and clinical studies have shown that the results of diagnostic and therapeutic strategies of TGFβ-targeted antibody drugs, including nuclide-coupled drugs, are not satisfactory. Therefore, it is essential to find and develop new TGFβ-targeting small-molecule or peptide inhibitors, as well as diagnostic and therapeutic radionuclide-labeled conjugate drugs. According to distribution characteristics of TGFβ *in vivo* and the penetration of TGFβ-targeted carrier drugs into solid tumors (1, 31, 37), radionuclides such as ^18^F and ^68^Ga can be used to label TGFβ-targeted small molecules or peptides, for research and application development of diagnostic drugs, and the potential indications may include but not be limited to hematological malignancies, sarcomas, pancreatic adenocarcinoma, and lung squamous cell carcinoma.

## 5 Conclusions

Conclusively, we found that the radiolabeled TGFβ-targeting tracer [^68^Ga]Ga-P144 has good microPET imaging efficiency, high target specificity, and tumor-targeting effect. As a molecular imaging tracer, [^68^Ga]Ga-P144 and its homologs may be applied as a promising diagnostic tool for TGFβ-positive tumors. TGFβ-targeting peptide inhibitors such as P144 and the labeled radiopharmaceuticals have potential theranostic value for malignant tumors such as pancreatic cancer and glioblastoma. In addition, investigations with PET imaging tracers targeting the TGFβ signaling pathway will be helpful and lead to the research and findings of new radiolabeled or conjugated drugs for the theranostics of TGFβ-positive malignancies.

## Data availability statement

The original contributions presented in the study are included in the article/Supplementary Material. Further inquiries can be directed to the corresponding authors.

## Ethics statement

The animal study was approved by the Institutional Animal Care and Research Ethics Committee of Shenzhen Hospital of Southern Medical University. The study was conducted in accordance with the local legislation and institutional requirements.

## Author contributions

YL conceived the study, performed the experiments, and wrote the manuscript. HZ analyzed the data and revised the manuscript. SH, XZ, and HC performed the experiments and helped with the methodology. QZ gave useful suggestions and discussed and revised the manuscript. All authors contributed to the article and approved the submitted version.
